# Seasonal variations of the prevalence of metabolic syndrome and its markers using big-data of health check-ups

**DOI:** 10.1265/ehpm.23-00216

**Published:** 2024-01-20

**Authors:** Hiroe Seto, Hiroshi Toki, Shuji Kitora, Asuka Oyama, Ryohei Yamamoto

**Affiliations:** 1 Graduate School of Human Sciences, Osaka University, Osaka 565-0871, Japan; 2 Health Care Division, Health and Counseling Center, Osaka University, Osaka 560-0043, Japan; 3 Research Center for Nuclear Physics, Osaka University, Osaka 567-0047, Japan; 4 Laboratory of Behavioral Health Promotion, Department of Health Promotion, Graduate School of Medicine, Osaka University, Osaka 565-0043, Japan

**Keywords:** Metabolic syndrome, Seasonal variation, Seasonal trend decomposition, STL, Specific health checkups

## Abstract

**Background:**

It is crucial to understand the seasonal variation of Metabolic Syndrome (MetS) for the detection and management of MetS. Previous studies have demonstrated the seasonal variations in MetS prevalence and its markers, but their methods are not robust. To clarify the concrete seasonal variations in the MetS prevalence and its markers, we utilized a powerful method called Seasonal Trend Decomposition Procedure based on LOESS (STL) and a big dataset of health checkups.

**Methods:**

A total of 1,819,214 records of health checkups (759,839 records for men and 1,059,375 records for women) between April 2012 and December 2017 were included in this study. We examined the seasonal variations in the MetS prevalence and its markers using 5 years and 9 months health checkup data and STL analysis. MetS markers consisted of waist circumference (WC), systolic blood pressure (SBP), diastolic blood pressure (DBP), triglycerides (TG), high-density lipoprotein cholesterol (HDL-C), fasting plasma glucose (FPG).

**Results:**

We found that the MetS prevalence was high in winter and somewhat high in August. Among men, MetS prevalence was 2.64 ± 0.42 (mean ± SD) % higher in the highest month (January) than in the lowest month (June). Among women, MetS prevalence was 0.53 ± 0.24% higher in the highest month (January) than in the lowest month (June). Additionally, SBP, DBP, and HDL-C exhibited simple variations, being higher in winter and lower in summer, while WC, TG, and FPG displayed more complex variations.

**Conclusions:**

This finding, complex seasonal variations of MetS prevalence, WC, TG, and FPG, could not be derived from previous studies using just the mean values in spring, summer, autumn and winter or the cosinor analysis. More attention should be paid to factors affecting seasonal variations of central obesity, dyslipidemia and insulin resistance.

**Supplementary information:**

The online version contains supplementary material available at https://doi.org/10.1265/ehpm.23-00216.

## Background

Recently, numerous studies have revealed that MetS can lead to a variety of serious conditions beyond cardiovascular disease and diabetes, including non-alcoholic fatty liver disease [1], chronic kidney disease [2], many cancer types [3], and even Parkinson’s disease [4]. MetS is composed of central obesity, hypertension, dyslipidemia, and insulin resistance, and the diagnosis uses the following six markers (MetS markers): waist circumference (WC), systolic blood pressure (SBP) and diastolic blood pressure (DBP), triglycerides (TG), high-density lipoprotein cholesterol (HDL-C), and fasting plasma glucose (FPG) [5]. Given that the improvement of MetS markers can prevent serious non-infectious diseases, screening and intervention programs for MetS have been introduced in many countries. In particular, in Japan, the law urges all adults between the ages of 40 and 74 to participate in the Specific Health Checkups and Specific Health Guidance every year, a program to screen for metabolic syndrome and provide health guidance [6].

MetS and its markers are known to have seasonal variations [7, 8]. For example, many papers are reporting that blood pressure rises in winter [9]. Many papers have also concluded that central obesity, dyslipidemia, and insulin resistance exhibit seasonal variations [10–13]. Some studies also investigated the factors behind seasonal variation. Blood pressure is said to depend on outside temperature [14, 15]. There are reports that the seasonal variation of MetS is associated with seasonal changes in mood and behavior [16, 17]. In addition, some papers reported that weight and blood glucose were affected by holidays and festivals [18, 19]. To effectively manage MetS, it is crucial to have a good understanding of the seasonal variations in MetS prevalence and MetS markers.

However, the problem is that it remains unclear how MetS prevalence and its markers change over the course of a year. Although some papers compared seasonal or monthly means of MetS prevalence or its markers [7, 20, 21], these results may not be representative of seasonal variations, because the results include abrupt changes or secular trends [22]. Cosinor analysis has also been used in some studies to model seasonal variations [12, 23, 24]. However, their results may not be able to model actual seasonal variations, because the seasonal variations of MetS prevalence and MetS markers can be more complex than sinusoidal [22]. To better understand the seasonal variation of MetS and its factors, we need to carefully observe the seasonal variations of MetS prevalence and its markers, excluding abrupt changes and secular trends, without assuming a simple model.

Against this background, the objective of this study was to clarify the concrete seasonal variations in the MetS prevalence and its markers. We analyzed the seasonal variations using the Seasonal Trend Decomposition Procedure based on LOESS (STL) [25]. STL can decompose time series data into the secular trend, seasonal variation, and residual without assuming a simple mathematical model. As for data, we utilized the Specific Health Checkups data from Osaka, Japan, containing about 2 million health checkup records.

## Methods

### Study population

We investigated the seasonal variations of MetS prevalence and its markers among Japanese adults attending the Specific Health Checkups program. This study used the Specific Health Checkups data from the Kokuho-database (KDB) of Osaka, Japan. The KDB consists of the National Health Insurance data and Senior Elderly Insurance data in the Osaka prefecture, Japan. Osaka has a population of approximately 8 million people, of which around 2 million insured individuals are recorded in the KDB each year. We included participants taking the Specific Health Checkups from April 1, 2012 to December 31, 2017, who did not currently take any medications for diabetes, hypertension and dyslipidemia (based on self-reports mentioning that they were receiving these medications). Our study protocol was approved by the Ethics Committee of Health and Counseling Center, Osaka University (IRB Approval Number 2022-7). All procedures involving human participants were conducted per the 1964 Declaration of Helsinki and its later amendments or comparable ethical standards. Informed consent was not obtained from participants because all data were anonymized according to the Japanese Ethical Guidelines for Medical and Health Research Involving Human Subjects enacted by the Ministry of Health, Labor, and Welfare of Japan.

### Study location and meteorological trends

Osaka is at 135° east longitude, 35° north latitude. It belongs to the climate zone with a humid subtropical climate (Köppen climate classification Cfa) characterized by four distinct seasons. It has very hot and humid summers (temperatures exceed 30 degrees Celsius) and moderately cold and dry winters (temperatures rarely drop below zero). Rainy days are rare compared to other areas in Japan. The heat island phenomenon is intense in the city center, and the nighttime temperature does not easily drop. In winter, there is almost no snow on the plains. The detail meteorological information of Osaka from April 1, 2012 to December 31, 2017 is shown in Supplementary Fig. 1.

### Subject selection

In this study, we used the Specific Health Checkups data of Osaka prefecture from April 1, 2012 to December 31, 2017, and 3,721,224 records from 1,398,335 individuals were available. We excluded 16 subjects that did not have consistent sex or birthday. We deleted the older records when there were two or more records for the same individual in the same fiscal year (9,230 records). We also removed those who currently had medications for diabetes, hypertension or dyslipidemia or they did not have self-reports about these medications (613,369 subjects). As a result, we analyzed 1,819,214 records from 784,950 individuals. The flowchart describing the subject selection is shown in Supplementary Fig. 2.

### MetS markers

In this study, we analyzed MetS markers; waist circumference (WC) as a central obesity indicator, systolic blood pressure (SBP) and diastolic blood pressure (DBP) as hypertension indicators, triglycerides (TG) and high-density lipoprotein cholesterol (HDL-C) as dyslipidemia indicators, and fasting plasma glucose (FPG) as an insulin resistance indicator.

Health checkup agencies were mandated to guarantee adequate internal and external quality control of measurements in their laboratories, as stipulated in the notification from the Ministry of Health, Labour and Welfare [26]. Waist circumference was measured by health professionals to the nearest millimeter at the level of the umbilicus in an upright position after a light exhalation. SBP and DBP were recorded once or twice at each specific health checkup for each subject. To ensure measurement consistency across subjects, we were able to use the first measurement of blood pressures, or the second measurement if the first was invalid. TG and HDL-C were measured using visible or ultraviolet spectrophotometric methods. FPG was measured after fasting for at least 10 hours. Blood was centrifuged within 6 hours of blood collection and measured within 72 hours. Measurement methods include potentiometric method, visible absorption photometry, and ultraviolet absorption photometry.

Abnormal values such as 0 or 9.999 and outliers within 0.005 percentiles from both the bottom and top values were not used for analyses.

Furthermore, we also investigated the prevalence of MetS. MetS was defined according to the Japanese Committee of the Criteria for MetS (JCCMS) [27]. In its guideline, MetS was defined central obesity (in men and women with WC of ≥85 cm and ≥90 cm respectively) and 2 of the following 3 factors: (a) hypertension (SBP ≥ 130 mmHg and/or DBP ≥ 85 mmHg), (b) dyslipidemia (TG ≥ 150 mg/dL and/or HDL-C < 40 mg/dL), (c) insulin resistance (FPG ≥ 110 mg/dL). Also, for each of WC, SBP, DBP, TG, HDL-C and FPG, we investigated the prevalences of individuals whose markers were out of the standard range.

In addition, we calculated the seasonal variation of the prevalence of MetS defined according to the Asian diagnostic criteria [5]. According to that, MetS is defined as the presence of at least three of the following five risk factors: (1) WC ≥ 90 cm (men), WC ≥ 80 cm (women); (2) TG ≥ 150 mg/dL; (3) HDL-C < 40 mg/dL (men), HDL-C < 50 mg/dL (women); (4) SBP ≥ 130 mmHg and/or DBP ≥ 85 mmHg; and (5) FPG ≥ 100 mg/dL. Detailed criteria and the results are provided in the Supplementary material in the section “Seasonal variations of the prevalence of MetS defined according to the Asian diagnostic criteria”.

### Statistical analysis

#### Seasonal trend decomposition procedure based on LOESS

For seasonal analyses, we used Seasonal Trend Decomposition Procedure based on LOESS (STL), which is one of the Seasonal Trend Decomposition methods [22]. The STL method assumes that time series data can be decomposed into the secular trend, seasonal variation, and residual component as shown in Equation 1.

$$Y_{t}=T_{t}+S_{t}+R_{t}\;(t=1,2,\ldots ,n).$$ (1)

Here, *t* = 1, 2, … *n* denotes the time, *Y_t_* denotes observed data, *T_t_* denotes the secular trend component, *S_t_* denotes the seasonal variation component, and *R_t_* denotes the residual component. The residual component *R_t_* is assumed to follow a normal distribution with mean 0 and constant variance, and its autocorrelation is always assumed to be 0 after removing the variance. Additionally, an important constraint to ensure the orthogonality of the secular trend and seasonal variation is that the seasonal component *S_t_* (*t* = 1, 2, … , *n*) is centered around zero on average $\sum_{t=1}^{n}S_{t}=0$ .

STL numerically decomposes the secular trend, seasonal variation and residual using a locally weighted regression method (LOESS). LOESS can derive secular trend and seasonal variation from complex data separately from residual. In our study, the variable is just year-month (such as May, 2012) and the observed value (such as the prevalence of MetS in May, 2012), so let *x_i_* (*i* = 1, 2, … , *n*) be year-month, and let *y_i_* (*i* = 1, 2, … , *n*) be observed data. In STL, LOESS performs a locally weighted linear regression on each point *x_c_*. The loss function of LOESS $L(\beta_{c0},\beta_{c1})$ is defined using the weighting function $W(x_{c},x_{i})$ as follows:

$$L(\beta_{c0},\beta_{c1})=\sum_{i=1}^{n}W(x_{c},x_{i})(y_{i}-(\beta_{c0}+\beta_{c1}x_{i}){)}^{2}.$$ (2)

This local regression is performed for all data points *c* = 1, … *n*, and the outputs ${\hat{y}}_{c}$ (*c* = 1, 2, … , *n*) are obtained from *β_c_*_0_ + *β_c_*_1_*x_i_*.

The weight function $W(x_{c},x_{i})$ consists of two functions, $W(x_{c},x_{i})=A(x_{c},x_{i})B(R_{x_{i}})$ , where

$$A(x_{c},x_{i})=\text{1}\;\text{l}\left(0≦\frac{|x_{c}-x_{i}|}{d_{c,q}}<1\right){\left(1-{\left(\frac{|x_{c}-x_{i}|}{d_{c,q}}\right)}^{3}\right)}^{3},$$ (3)

$$B(R_{x_{i}})=\text{1}\;\text{l}\left(0≦\frac{|R_{x_{i}}|}{6R_{\text{med}}}<1\right){\left(1-{\left(\frac{|R_{x_{i}}|}{6R_{\text{med}}}\right)}^{2}\right)}^{2}.$$ (4)

Here, 1l(a) is an indicator function to return 1 when *a* is true, and 0 otherwise. *d_c_*_,_*_q_* is the distance from the point *x_c_* to the *q*-th farthest point from *x_c_*. $R_{x_{i}}$ is the residual at *x_i_* and *R_med_* is the median of all residual *R*. *A*(*x_c_*, *x_i_*) gives greater weight to point *x_i_* as *x_i_* is closer to *x_c_*, as long as *x_i_* is within distance *d_c_*_,_*_q_* from *x_c_*. $B(R_{x_{i}})$ gives greater weight to point *x_i_* as $R_{x_{i}}$ is smaller, as long as $R_{x_{i}}$ is smaller than 6*R_med_*.

STL utilizes LOESS to numerically derive the secular trend and seasonal variation and is composed of two nested recursive loops, inner and outer loops. In the inner-loop, LOESS is used to repeatedly update the secular trend component *T_t_* and the seasonal variation component *S_t_*. After the *k*-th loop, the values of the seasonal variation component $S_{t}^{k+1}$ and secular trend component $T_{t}^{k+1}$ are obtained through the following six steps:

**Step 1** *Detrending:* A detrended series ${﻿}^{**}S_{t}^{k+1}=Y_{t}-T_{t}^{k}$ is computed.
**Step 2** *Cycle-subseries smoothing:* Define cycle-subseries as a group of data points in the same month in various years. In this study, one of the cycle-subseries was {2012/4, 2013/4, 2014/4, 2015/4, 2016/4, 2017/4}. Let *n_p_* be the number of cycle-subseries, then *n_p_* = 12 in this study. Execute LOESS for each cycle-subseries and obtain the output ${﻿}^{*}S_{t}^{k+1}$ .
**Step 3** *Low-pass filtering of smoothing cycle-subseries:* The low-pass filter is to be constructed by applying moving average three times and LOESS on ${﻿}^{*}S_{t}^{k+1}$ to obtain smoothed secular component. The resulting component is named $L_{i}^{k+1}$ , which is very smooth.
**Step 4** *Detrending of smoothing cycle-subseries:* The seasonal component of *k* + 1-th loop is calculated by $S_{t}^{k+1}{=}^{*}S_{t}^{k+1}-L_{t}^{k+1}$ .
**Step 5** *Deseasonalising:* A deseasonalized series ${﻿}^{*}T_{i}^{k+1}=Y_{t}-S_{t}^{k+1}$ is computed.
**Step 6** *Trend smoothing:* $T_{t}^{k+1}$ is obtained by LOESS smoothing of the deseasonalized series ${﻿}^{*}T_{i}^{k+1}$ .

In the outer loop, the residual component is renewed from $R_{i}^{k}$ to $R_{i}^{k+1}$ by calculating $R_{i}^{k+1}=Y_{i}-T_{i}^{k+1}-S_{i}^{k+1}$ . These $R_{i}^{k+1}$ are then used for LOESS of the next step of the inner loop to obtain converged values. STL extracts the secular trend and seasonal variation from the data by recursively iterating the smoothing process. It is robust to extracting the secular trend and seasonal variation and can decompose time-series data even for a dataset with missing values [22].

In this study, we used the package of the Python package: statsmodels.tsa.seasonal.STL.

#### Statistical analysis

All analyses were stratified by sex. We first calculated the descriptive statistics for age and the six MetS markers for each season and sex. Each season was classified as follows: spring (March to May), summer (June to August), autumn (September to November) and winter (December to February). Variables were described with median and interquartile range (IQR) (25th and 75th percentile).

We used STL, as described above, to investigate the seasonal variations of MetS prevalence, the prevalences of individuals whose markers were out of the standard range, and MetS markers. To confirm that STL can extract the secular trend and seasonal variation from six years of the observation data, we plotted three components: the observed data, the secular trend and the seasonal variation. As the observed data and secular trends, we plotted the monthly means from April 2012 to December 2017. The seasonal variation was calculated by removing the secular trend and residual from the observed data. Then for each month, we calculated the means and standard deviations (SD) of the seasonal variation from five or six years of data. Additionally, we calculated the differences between the month with the highest mean and the month with the lowest mean in each year and the mean and SD of those differences. And we plotted the seasonal variation results from January to December for each year and plotted the mean and SD for each month.

In addition, regarding the seasonal variations of MetS markers, further stratified analyses were conducted with the following four groups: middle-aged men (<65 years of age), middle-aged women (<65 years of age), elderly men (≥65 years of age), elderly women (≥65 years of age).

## Results

### Characteristics of subjects

1,819,214 records (759,839 records for men and 1,059,375 records for women) were included in this study, with the overall median age being 67.0 years old. Table 1 shows the characteristics of subjects for each sex and season. WC was highest in spring and winter among men, with a median of 83.5 cm. SBP and DBP were lowest in summer among both men and women, with a respective median (men/women) of 124.0/122.0 mmHg and 72.0/70.0 mmHg. TG was highest in summer among both men and women, with a median (men/women) of 92.0/86.0 mg/dL. HDL-C was lowest in summer among both men and women, with a median (men/women) of 61.0/66.0 mg/dL. FPG was highest in winter among men, with a median of 95.0 mg/dL.

**Table 1:** The characteristics of subjects for each season and sex. Values are presented as median [Q1, Q3]. Abbreviations: WC, waist circumference; SBP, systolic blood pressure; DBP, diastolic blood pressure; TG, triglycerides; HDL-C, high-density lipoprotein cholesterol; FPG, fasting plasma glucose.

	**Spring (Mar–May)**	**Summer (Jun–Aug)**	**Autumn (Sep–Nov)**	**Winter (Dec–Feb)**
Men				
Overall subjects, n	178,970	226,146	217,704	137,019
Subjects of 2012, n	14,375	37,117	36,346	7,809
Subjects of 2013, n	32,380	37,529	36,627	26,655
Subjects of 2014, n	32,742	39,119	37,932	26,520
Subjects of 2015, n	32,937	38,823	37,685	27,687
Subjects of 2016, n	33,001	37,721	37,142	26,698
Subjects of 2017, n	33,535	35,837	31972	21,650
Age (years)	67.0 [56.0,74.0]	67.0 [54.0,73.0]	68.0 [58.0,74.0]	66.0 [54.0,72.0]
WC (cm)	83.5 [78.0,89.0]	83.0 [78.0,88.5]	83.1 [78.0,88.5]	83.5 [78.0,89.0]
SBP (mmHg)	128.0 [118.0,140.0]	126.0 [114.0,137.0]	128.0 [117.0,140.0]	129.0 [118.0,140.0]
DBP (mmHg)	77.0 [70.0,84.0]	75.0 [68.0,82.0]	77.0 [70.0,84.0]	78.0 [70.0,85.0]
TG (mg/dL)	101.0 [72.0,146.0]	102.0 [73.0,149.0]	100.0 [72.0,144.0]	100.0 [72.0,145.0]
HDL-C (mg/dL)	57.0 [48.0,68.0]	55.0 [46.0,66.0]	57.0 [48.0,68.0]	58.0 [48.0,69.0]
FPG (mg/dL)	94.0 [88.0,103.0]	94.0 [88.0,101.0]	94.0 [88.0,101.0]	95.0 [89.0,103.0]

Women				
Overall subjects, n	238,283	286,697	334,314	200,081
Subjects of 2012, n	16,483	47,337	54,897	11,346
Subjects of 2013, n	43,742	47,412	55,743	38,696
Subjects of 2014, n	44,530	49,518	57,723	38,750
Subjects of 2015, n	43,991	49,324	58,409	40,591
Subjects of 2016, n	43,829	47,600	57,378	38,958
Subjects of 2017, n	45,708	45,506	50,164	31,740
Age (years)	66.0 [58.0,73.0]	66.0 [57.0,73.0]	67.0 [59.0,73.0]	65.0 [56.0,71.0]
WC (cm)	78.0 [72.0,85.0]	78.0 [72.0,84.5]	78.0 [72.0,84.5]	78.0 [72.0,84.6]
SBP (mmHg)	124.0 [112.0,136.0]	122.0 [110.0,134.0]	124.0 [112.0,136.0]	124.0 [112.0,136.0]
DBP (mmHg)	72.0 [65.0,80.0]	70.0 [64.0,79.0]	72.0 [65.0,80.0]	72.0 [66.0,80.0]
TG (mg/dL)	85.0 [63.0,116.0]	86.0 [64.0,118.0]	84.0 [63.0,116.0]	82.0 [61.0,112.0]
HDL-C (mg/dL)	69.0 [58.0,81.0]	66.0 [56.0,78.0]	69.0 [58.0,80.0]	70.0 [59.0,82.0]
FPG (mg/dL)	90.0 [85.0,97.0]	90.0 [85.0,96.0]	90.0 [85.0,96.0]	90.0 [85.0,97.0]

### Secular trend and seasonal variation of MetS prevalence

**Fig. 1:**
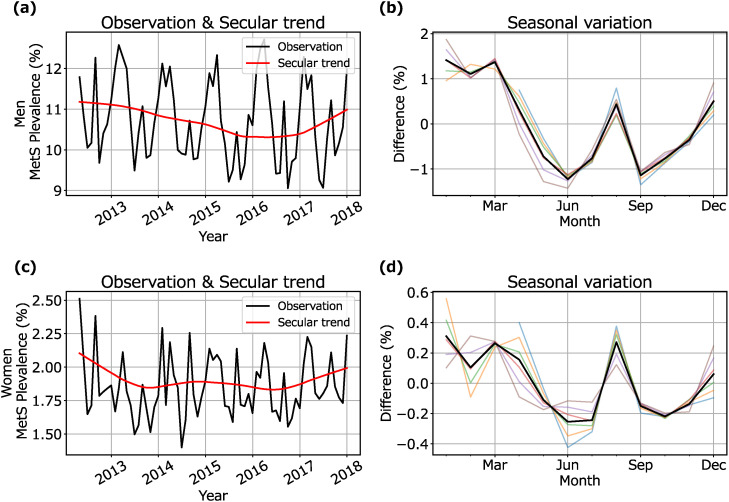
Secular trend and seasonal variation in MetS prevalence: In the two figures on the left, the black lines represent the observed data and the red lines represent the secular trends. In the two figures on the right, thin lines in blue, orange, green, red, purple, and brown show the results of seasonal variations in 2012, 2013, 2014, 2015, 2016, and 2017, respectively. The thick black lines show the 6-year average values.

Figure 1 shows the observed data, secular trends, and seasonal variations for the MetS prevalence of men (upper) and women (lower). The observed data showed multiple increases and decreases throughout the year. The secular trends were highest for both men and women in April 2012, at 11.18% and 2.10%, respectively. The MetS prevalence was high in winter for both men and women. Interestingly, the MetS prevalence in August was also high among both men and women, despite being summer. Among men, MetS prevalence was 2.64 ± 0.42 (mean ± SD) % higher in the highest month (January) than in the lowest month (June). Among women, MetS prevalence was 0.53 ± 0.24% higher in the highest month (January) than in the lowest month (June).

**Fig. 2:**
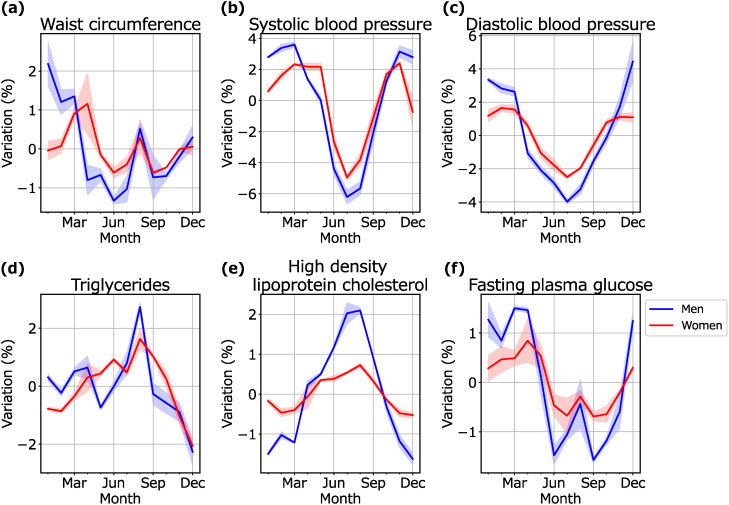
Seasonal variations in the prevalence of individuals whose markers were out of the standard range: The blue lines and blue shaded areas represent the means and SD of seasonal variations for each year for men. The red lines and red shaded areas represent the means and SD of seasonal variations for each year for women.

Figure 2 shows the seasonal variations of the prevalences of individuals whose markers were out of the standard range. The results of SBP, DBP and HDL-C showed simple variations. The prevalences of individuals whose SBP and DBP were out of the standard range were low in summer, and the result of HDL-C was high in summer. On the other hand, the results of WC, TG and FPG showed complex variations. The prevalences of individuals whose WC and FPG were out of the standard range were low from summer to Autumn except for August, and that of TG was remarkably high in August, which could be the reason for the high prevalence of MetS in August. The differences between the highest month and the lowest month (men/women) were 3.49 ± 0.53%/1.77 ± 0.80% for WC, 9.94 ± 0.57%/7.35 ± 0.20% for SBP, 8.42 ± 1.32%/4.11 ± 0.22% for DBP, 5.00 ± 0.43%/3.70 ± 0.20% for TG, 3.72 ± 0.21%/1.25 ± 0.10% for HDL-C, and 3.05 ± 0.05%/1.53 ± 0.52% for FPG. The observed data and secular trends of the prevalences of individuals whose markers were out of the standard range are shown in Supplementary Fig. 3 (men) and Supplementary Fig. 4 (women).

### Seasonal variation of MetS markers

The results of seasonal variations in MetS markers are shown in Supplementary Fig. 5. The observed data and secular trends of MetS markers are shown in Supplementary Fig. 6 (men) and Supplementary Fig. 7 (women). Similarly to the seasonal variations of the prevalences of individuals whose markers were out of the standard range, the results of SBP, DBP and HDL-C showed simple variations and the results of WC, TG and FPG showed complex variations. The differences between the highest month and the lowest month (men/women) were 0.49 ± 0.09 (mean ± SD) cm/0.42 ± 0.05 cm for WC, 4.44 ± 0.16 mmHg/3.47 ± 0.17 mmHg for SBP, 3.14 ± 0.52 mmHg/2.31 ± 0.03 mmHg for DBP, 9.18 ± 0.74 mg/dL/10.35 ± 0.19 mg/dL for TG, 3.93 ± 0.37 mg/dL/4.64 ± 0.22 mg/dL for HDL-C, and 1.92 ± 0.42 mg/dL/1.10 ± 0.13 mg/dL for FPG.

### Stratified analysis

**Fig. 3:**
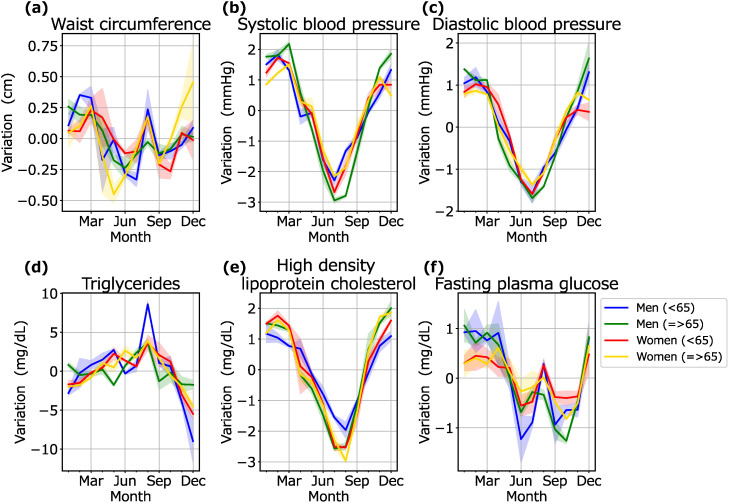
Seasonal variations in MetS markers stratified by sex and age: The blue lines and blue shaded areas represent the means and SD of seasonal variations for each year for middle-aged men. The green lines and green shaded areas represent the means and SD of seasonal variations for each year for elderly men. The red lines and red shaded areas represent the means and SD of seasonal variations for each year for middle-aged women. The yellow lines and yellow shaded areas represent the means and SD of seasonal variations for each year for elderly women.

Figure 3 shows the seasonal variations for each MetS marker by sex and age group. For almost all markers, the seasonal variations showed almost the same patterns across sex and age. All groups shared almost the same variations. For TG, significant peaks were observed especially in middle-aged men in April and August. For FPG, middle-aged men had the lowest value in June, and elderly men had the lowest value in October.

## Discussions

This study was conducted with the aim to clarify the concrete seasonal variations in MetS prevalence and its markers. The result of seasonal variation for MetS prevalence showed that it tends to be high in winter and August despite being summer. Moreover, we showed that the high prevalence in August could be attributed to the high WC, TG, HDL-C and FPG. Although we were unable to monitor health checkup data of each individual over long period, the results of seasonal variations in some markers align with the results of previous longitudinal monitoring studies. For example, the seasonal variations for SBP and DBP of this study are consistent with those of a study that monitored the daily home BP of 64,536 Japanese people for 2 years [28]. The result that WC was low from summer to autumn except August is similar to the results of two studies that monitored the daily body weight of Japanese people [18, 29]. Therefore, although we cannot deny the possibility that our results were due to differences in the subject characteristics in each month, those are likely to represent the individual seasonal variations of MetS markers.

As revealed by this study, the seasonal variations of MetS prevalence and its markers did not follow a sinusoidal wave, and some markers even had multiple peaks within a year. Furthermore, some markers have abrupt changes or secular trends. Previous studies did not deal with these problems because they relied on methods such as comparison of seasonal means [7, 20, 21, 30], cosinor analysis [12, 23, 24], and plotting monthly means [31, 32]. In contrast, our study succeeded in extracting complex seasonal variations using the robust analysis method of STL and big data from six years of health checkup data.

We found that MetS prevalence showed a complex variation and was high in winter and somewhat high in August. The main cause of this would be the fact that the prevalence of individuals whose WC were out of the standard range was high in winter and August, as central obesity is required for a diagnosis of MetS according to the JCCMS definition. In addition, the prevalences of individuals whose SBP and DBP were out of the standard range were higher than other markers, and their seasonal variations were also large, which may have a large impact on the high MetS prevalence in winter. Furthermore, the high prevalences of individuals whose TG, HDL-C and FBP were out of the standard range in August may have an impact on the high MetS prevalence in August. We also found that there are two types of MetS markers: those showing simple variations and those showing complex variations. SBP, DBP, and HDL-C exhibited simple variations. The reason might be that BP and HDL-C are strongly influenced by temperature [21, 28, 32, 33]. On the other hand, WC, TG, and FPG exhibited complex variations. WC and FPG were low in summer except for August, and TG was remarkably high in August. Such high values in August have been overlooked in previous studies [8, 24, 34]. Since only August shows a spike in summer, it is likely due to other factors other than temperature. One factor might be summer vacation or a festival in August. Various papers have reported that food intake increases and body weight and blood sugar levels rise during long holidays and festivals [18, 19, 29, 35, 36], and Japanese people traditionally take a long vacation around the *Obon festival* in August. Also, some papers reported that physical activity may help reduce the risk of MetS [37, 38], and the number of steps of Japanese people is the lowest in August [39], so seasonal variation of physical activity may also be one of the reasons. In any case, few studies have investigated the causes of high WC, TG, and FPG in August, so further research is needed. Furthermore, previous studies have shown that there are seasonal variations in intakes of energy, vegetable and fruit intake, and vitamin C contained in fruits and vegetables [40–42]. Also, because the difference between body and air temperature is slight in summer, the metabolic rate may be lower in summer than in winter. These factors may influence the seasonal variation of MetS and its markers.

From the results of this study, it has been shown that MetS prevalence changed by 2.64 ± 0.42 (mean ± SD) % among men and 0.53 ± 0.24% among women within a year. So due to this seasonal variation, the month in which health checkups are conducted may affect the clinical diagnosis and management of MetS. In some cases, it could lead to the oversight of hypertension, dyslipidemia, and diabetes, which are important public health concerns [43]. On the other hand, excessive interventions could result in unnecessary healthcare expenditures. Here, the important question is in which month we can make the most reliable prognosis prediction, although there is limited research that tackles this question. Thus, in the future, we intend to explore when people should undergo health checkups or consider methods to adjust marker values.

Furthermore, the results of this study can have important implications for research concerning MetS markers. Research using MetS markers is being conducted around the world [44–47]. In these studies, it is important to address the seasonal variation effectively to prevent them from influencing the analysis results [22]. We believe that this study demonstrates the usefulness of the STL method as a way to model seasonal variation.

It should be noted that this study has several limitations. First, the results of seasonal variations of MetS prevalence and its markers might be due to differences in the characteristics of the subjects in each month. This is because people taking health checkups were able to decide when they took health checkups by themselves, and most people took their checkups in approximately the same month every year. Second, we removed those who had current medications for diabetes, hypertension and dyslipidemia, which might have affected the results. For example, antihypertensive drugs are more likely to be prescribed in the winter, thus people with high blood pressure might be more likely to be excluded in winter. These situations could have prevented us from correctly evaluating monthly prevalence. Third, we did not take into account socioeconomic status and the differences between group health checkups and individual health checkups, which might have influenced the results. For example, it has been reported that socioeconomic status influenced seasonal variation in blood pressure [15]. In addition, in Japan, there are individual health checkups and group checkups, and group checkups could cause bias in monthly age and socioeconomic status. Finally, since data from health checkups throughout Osaka were utilized, no prior agreement was established regarding quality control for the research. The biomarkers did not employ reagents from the same manufacturer. As health checkups were conducted inthe morning, afternoon, or evening, the potential impact of daily variations might have affected the results of this study.

## Conclusion

We found that the prevalence of MetS is high in winter, and also high in August despite being summer. The high prevalence in August can be attributed to the higher WC, TG, HDL-C and FPG. More attention should be paid to the seasonal factors of central obesity, dyslipidemia and insulin resistance.

## Abbreviations

MetS: metabolic syndrome
STL: Seasonal Trend Decomposition Procedure based on LOESS
WC: waist circumference
SBP: systolic blood pressure
DBP: diastolic blood pressure
TG: triglycerides
HDL-C: high-density lipoprotein cholesterol
FPG: fasting plasmaglucose
KDB: Kokuho-database
LOESS: locally weighted regression
IQR: interquartile range
SD: standard deviation

## Declarations

### Availability of data and material

Data cannot be shared publicly because of local governments own medical check-up data. Data are available from the Health and Counseling Center, Osaka University (contact via campuslifekenkou-syomu@hacc.osaka-u.ac.jp) for researchers who meet the criteriafor access to confidential data.
