# Supplementary material for: Seasonal variations of the prevalence of metabolic syndrome and its markers using big-data of health check-ups
Source: Environ Health Prev Med. 2024 Jan 20;29:2. doi: 10.1265/ehpm.23-00216 (PMC10808004; doi:10.1265/ehpm.23-00216)
Supplement: Supplementary file 1 — Additional file 1: Supporting Information. [file ehpm-29-002-s001.pdf]

Title

Supporting Information: Seasonal variations of the prevalence of metabolic syndrome and its markers using big-data of health check-ups

Author

Hiroe Seto[a,b], Hiroshi Toki[b,c], Shuji Kitora[b], Asuka Oyama[b], and Ryohei Yamamoto[b,d]

Institution

[a] Graduate School of Human Sciences, Osaka University, Osaka 565-0871, Japan

[b] Health Care Division, Health and Counseling Center, Osaka University, Osaka 560-0043, Japan

[c] Research Center for Nuclear Physics, Osaka University, Osaka 567-0047, Japan

[d] Laboratory of Behavioral Health Promotion, Department of Health Promotion, Graduate School of Medicine, Osaka University, Osaka 565-0043, Japan

Correspondence

H. Toki: toki@rcnp.osaka-u.ac.jp

This is supplementary material to the paper entitled "Seasonal variations of the prevalence of metabolic syndrome and its markers using big-data of health check-ups". Here, we provide several detailed backup pieces of information to assertions in the main text.

Study location and meteorological trends

Figure 1 shows the monthly means of daily mean temperature, precipitation, sunshine duration, daily mean pressure and daily mean humidity in Osaka from April 2012 to December 2017. The data were collected from the database of Japan Meteorological Agency [1]. The daily mean temperature ranges from around 6 ℃ in winter to 29 ℃ in summer. There is little precipitation in winter, and it increases significantly in June and autumn. Basically, there are few Sunshine duration in winter, but there is also little sunshine in June and autumn due to rainfall. The daily mean pressure appears a negative correlation to the daily mean temperature. The humidity reaches its peak in summer and remains high until autumn, while it becomes low during the winter to spring period.

Subject selection

The flowchart of the number of subjects is shown in supplementary Figure 2. 1,398,335 individuals received Specific Health Checkups from April 1, 2012 to December 31, 2017, and 3,721,224 records were included because these checkups can be taken up to once per person per year. 16 individuals (with 35 records) were excluded because they had inconsistencies in sex or birthday. Then we deleted the older records when there were two or more records for the same individual in the same fiscal year and it existed 9,230 records. Finally, we excluded 613,369 individuals (with 1,892,745 records) who self-reported that they received one or more medications for diabetes, hypertension and dyslipidemia or did not have

self reports. 1,819,214 records from 784,950 individuals are used for analysis. Approximately 300,000 individuals received the checkups within each fiscal year.

Secular trend of the prevalences of individuals whose markers were out of the standard range

The observed data and secular trends for the prevalences of individuals whose markers were out of the standard range are shown in Figure 3 (men) and Figure 4 (women).

Secular trend and seasonal variation of MetS markers

The results of seasonal variations for MetS markers are shown in Figure 5. The observed data and secular trends for MetS markers are shown in Figure 6 (men) and Figure 7 (women).

Seasonal variations of the prevalence of MetS defined according to the Asian diagnostic criteria

We defined MetS according to the Asian diagnostic criteria [2] and investigated the seasonal variations of MetS prevalence and the prevalences of individuals whose markers were out of the standard range using STL. According to the Asian diagnostic criteria, MetS is defined as the presence of at least three of the following five risk factors: (1) Elevated WC (WC $\geq$90 cm for men and WC $\geq$80 cm for women); (2) Elevated TG (TG $\geq$ 150 mg/dL); (3) Reduced HDL-C (HDL-C $<$ 40

mg/dL for men and HDL-C < 50 mg/dL for women); (4) Elevated blood pressure (SBP ≥ 130 mmHg and/or DBP ≥ 85 mmHg); and (5) Elevated fasting glucose (FPG ≥ 100 mg/dL). The thresholds are different between the Asian diagnostic criteria and JCCMS criteria for the following three risk factors: (1) WC (≥85 cm in the JCCMS criteria ≥90 cm in the Asian diagnostic criteria for men / ≥90 cm in the JCCMS criteria ≥80 cm in the Asian diagnostic criteria for women); (2) HDL-C (< 40 mg/dL in the JCCMS criteria <50 mg/dL in the Asian diagnostic criteria for women); (3) FPG (≥ 110 mg/dL in the JCCMS criteria ≥100 mg/dL in the Asian diagnostic criteria for both sex). Additionally, JCCMS mandates central obesity as a required risk factor, but there are no required risk factors in the Asian diagnostic criteria. Also, within the Asian diagnostic criteria, TG and HDL-C are treated as separate criteria.

Fig. 8 shows the observed data, secular trends, and seasonal variations for the MetS prevalence of men (upper) and women (lower). Fig. 9 shows the seasonal variations of the prevalences of individuals whose markers were out of the standard range. Because there are no required risk factors, MetS prevalence was about 10% higher for both men and women than the case of the JCCMS criteria. Accordingly, the differences between the highest month and the lowest month increased for both sex, and these were 3.49 ± 0.53% for men and 1.89 ± 0.22% for women. Among men, the plot of seasonal variation of MetS prevalence did not change significantly comparing to the case using the JCCMS criteria, probably because the difference between the Asian diagnostic criteria and the JCCMS criteria was small. Among women, MetS prevalence in January and February was lower than

the results calculated using the JCCMS criteria. The main reasons for this will be that the prevalence of individuals whose HDL-C was out of the standard range and seasonal variation of HDL-C have increased significantly. In fact, over the entire period of 5 years and 9 months, the prevalence of individuals whose HDL-C was out of the standard range was 1.67% using the JCCMS criteria, but 10.1% using the Asian diagnostic criteria. Furthermore, as seen in Fig. 9, the seasonal variation of HDL-C is extremely larger than the case using the JCCMS criteria. The differences between the highest month and the lowest month for HDL-C were 1.25 ± 0.10% in the case using the JCCMS criteria but 5.07± 0.16% in the case using the Asian diagnostic criteria.

References

1 (JMA) JMA. Historical Weather Database Download; 2023. https://www.data.jma.go.jp/obd/stats/etrn/index.php.

2 Alberti KGMM, Eckel RH, Grundy SM, Zimmet PZ, Cleeman JI, Donato KA, et al. Harmonizing the Metabolic Syndrome. Circulation. 2009 10;120:1640-5.

Legends of Figures

1 Meteorological trends: Monthly means values of daily mean temperature, precipitation, sunshine duration, daily mean pressure and daily mean humidity for the study period in Osaka City, Osaka Prefecture, Japan. 6
2 Flowchart of Subject. 7
3 Secular trends and observed data of the prevalence of individuals whose markers were out of the standard range among men: The black lines represent the observed data and the red lines represent the secular trends. 8
4 Secular trends and observed data of the prevalence of individuals whose markers were out of the standard range among women: The black lines represent the observed data and the red lines represent the secular trends. 9
5 Seasonal variations in MetS markers: The blue lines and blue shaded areas represent the means and SDs of seasonal variations for each year for men. The red lines and red shaded areas represent the means and SDs of seasonal variations for each year for women. 10
6 Secular trends and observed data of MetS markers among men: The black lines represent the observed data and the red lines represent the secular trends. 11
7 Secular trends and observed data of MetS markers among women: The black lines represent the observed data and the red lines represent the secular trends. 12
8 Seasonal variations of the prevalence of MetS defined according to the Asian diagnostic criteria: In the two figures on the left, the black lines represent the observed data and the red lines represent the secular trends. In the two figures on the right, thin lines in blue, orange, green, red, purple, and brown show the results of seasonal variations in 2012, 2013, 2014, 2015, 2016, and 2017, respectively. The thick black lines show the 6-year average values. 13
9 Seasonal variations in the prevalence of individuals whose markers were out of the standard range according to the Asian diagnostic criteria: The blue lines and blue shaded areas represent the means and SD of seasonal variations for each year for men. The red lines and red shaded areas represent the means and SD of seasonal variations for each year for women. 14

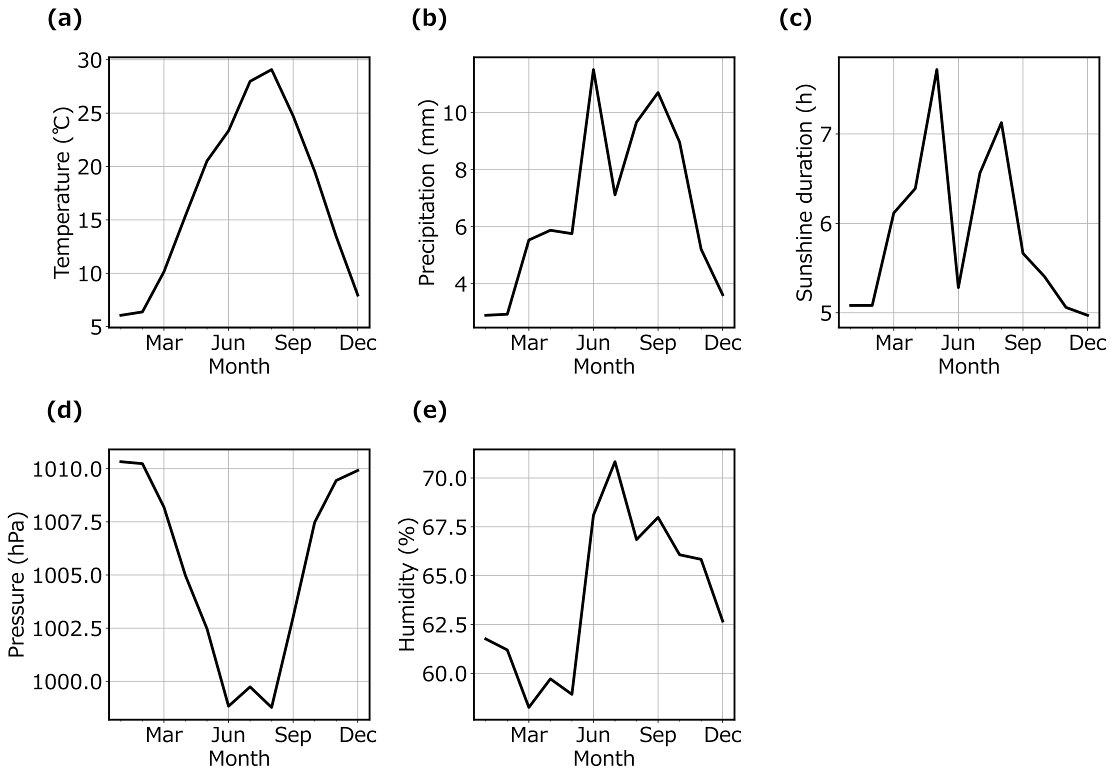
(a)
Temperature (℃)
30
25
20
15
10
5
Mar
Jun
Sep
Dec
Month
(b)
Precipitation (mm)
10
8
6
4
Mar
Jun
Sep
Dec
Month
(c)
Sunshine duration (h)
7
6
5
Mar
Jun
Sep
Dec
Month
(d)
Pressure (hPa)
1010.0
1007.5
1005.0
1002.5
1000.0
Mar
Jun
Sep
Dec
Month
(e)
Humidity (%)
70.0
67.5
65.0
62.5
60.0
Mar
Jun
Sep
Dec
Month

Fig. 1

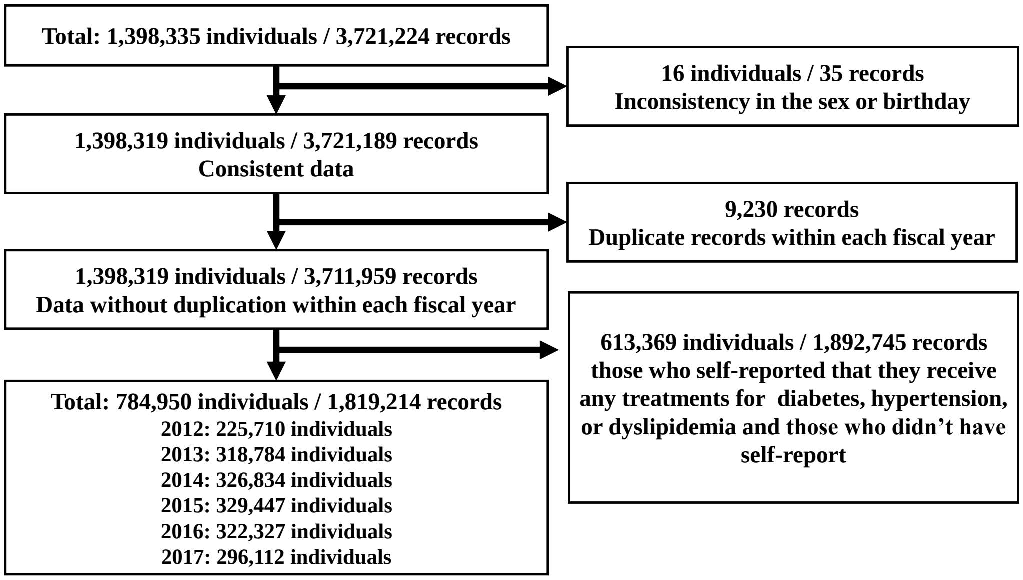

Total: 1,398,335 individuals / 3,721,224 records
16 individuals / 35 records
Inconsistency in the sex or birthday
1,398,319 individuals / 3,721,189 records
Consistent data
9,230 records
Duplicate records within each fiscal year
1,398,319 individuals / 3,711,959 records
Data without duplication within each fiscal year
613,369 individuals / 1,892,745 records
those who self-reported that they receive any treatments for diabetes, hypertension, or dyslipidemia and those who didn't have self-report
Total: 784,950 individuals / 1,819,214 records
2012: 225,710 individuals
2013: 318,784 individuals
2014: 326,834 individuals
2015: 329,447 individuals
2016: 322,327 individuals
2017: 296,112 individuals

Fig. 2

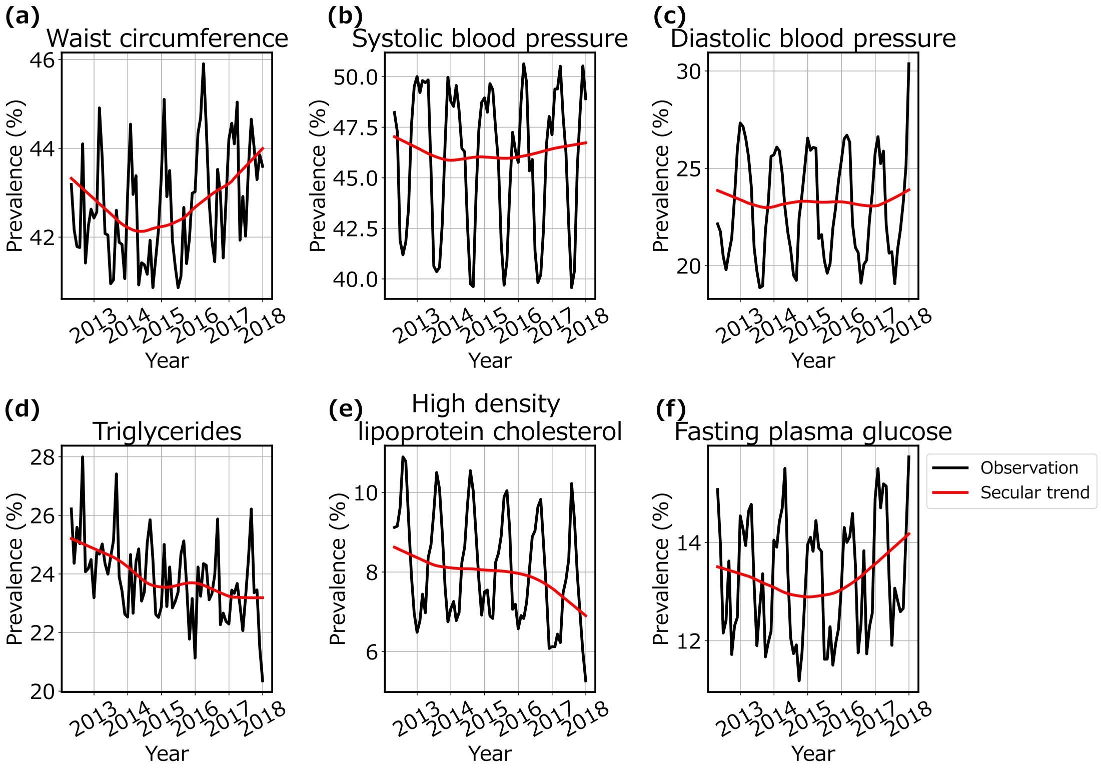
(a)
Waist circumference
Prevalence (%)
Year
(b)
Systolic blood pressure
Prevalence (%)
Year
(c)
Diastolic blood pressure
Prevalence (%)
Year
(d)
Triglycerides
Prevalence (%)
Year
(e)
High density
lipoprotein cholesterol
Prevalence (%)
Year
(f)
Fasting plasma glucose
Prevalence (%)
Year
Observation
Secular trend

Fig. 3

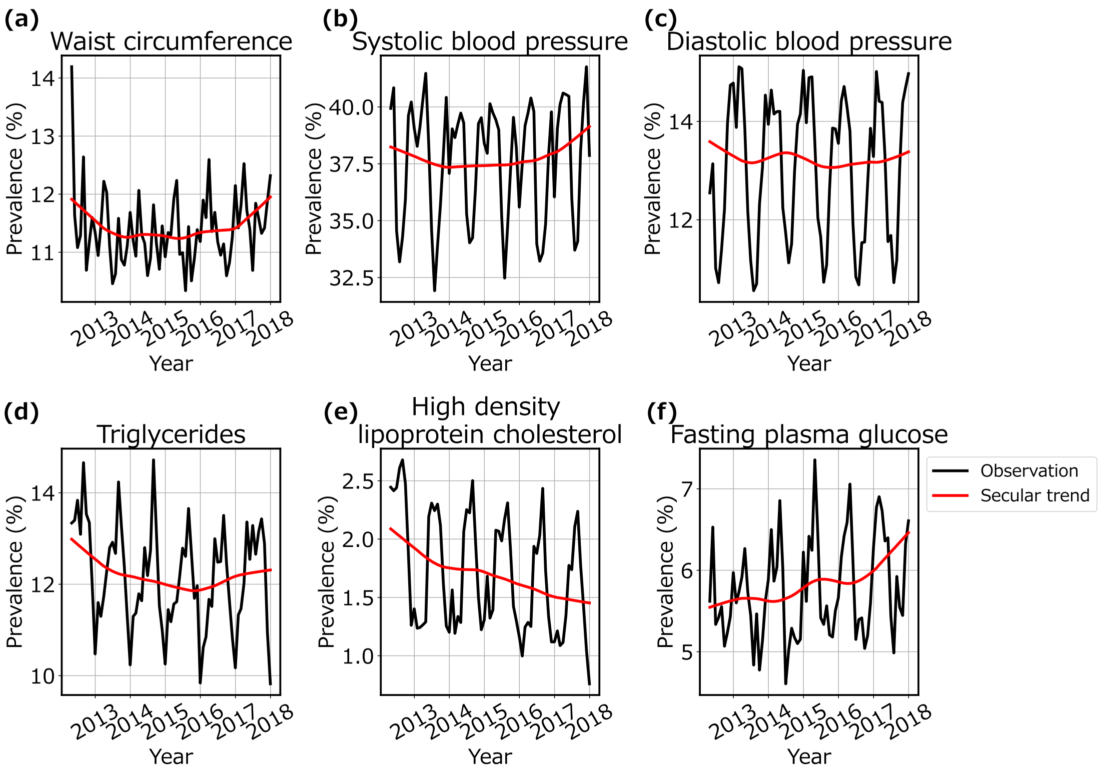
(a)
Waist circumference
Prevalence (%)
14
13
12
11
2013
2014
2015
2016
2017
2018
Year
(b)
Systolic blood pressure
Prevalence (%)
40.0
37.5
35.0
32.5
2013
2014
2015
2016
2017
2018
Year
(c)
Diastolic blood pressure
Prevalence (%)
14
12
2013
2014
2015
2016
2017
2018
Year
(d)
Triglycerides
Prevalence (%)
14
12
10
2013
2014
2015
2016
2017
2018
Year
(e)
High density
lipoprotein cholesterol
Prevalence (%)
2.5
2.0
1.5
1.0
2013
2014
2015
2016
2017
2018
Year
(f)
Fasting plasma glucose
Prevalence (%)
7
6
5
2013
2014
2015
2016
2017
2018
Year
Observation
Secular trend

Fig. 4

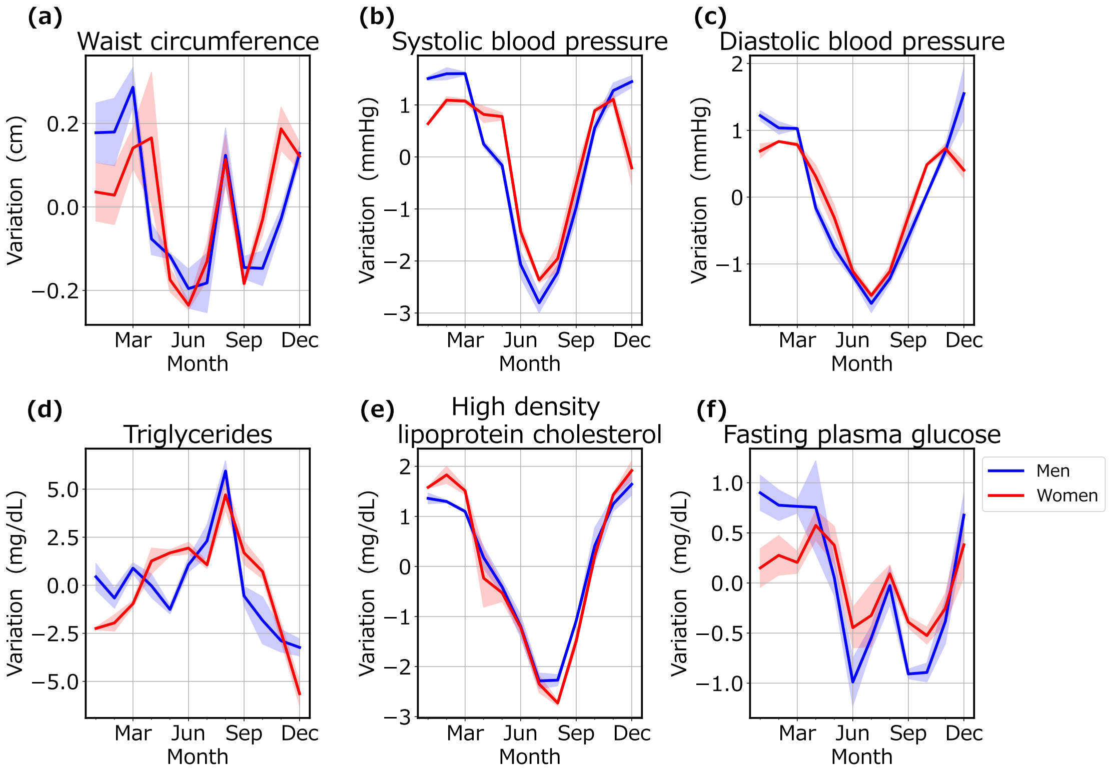
(a)
Waist circumference
Variation (cm)
Month
(b)
Systolic blood pressure
Variation (mmHg)
Month
(c)
Diastolic blood pressure
Variation (mmHg)
Month
(d)
Triglycerides
Variation (mg/dL)
Month
(e)
High density lipoprotein cholesterol
Variation (mg/dL)
Month
(f)
Fasting plasma glucose
Variation (mg/dL)
Month
Mar Jun Sep Dec
Men
Women

Fig. 5

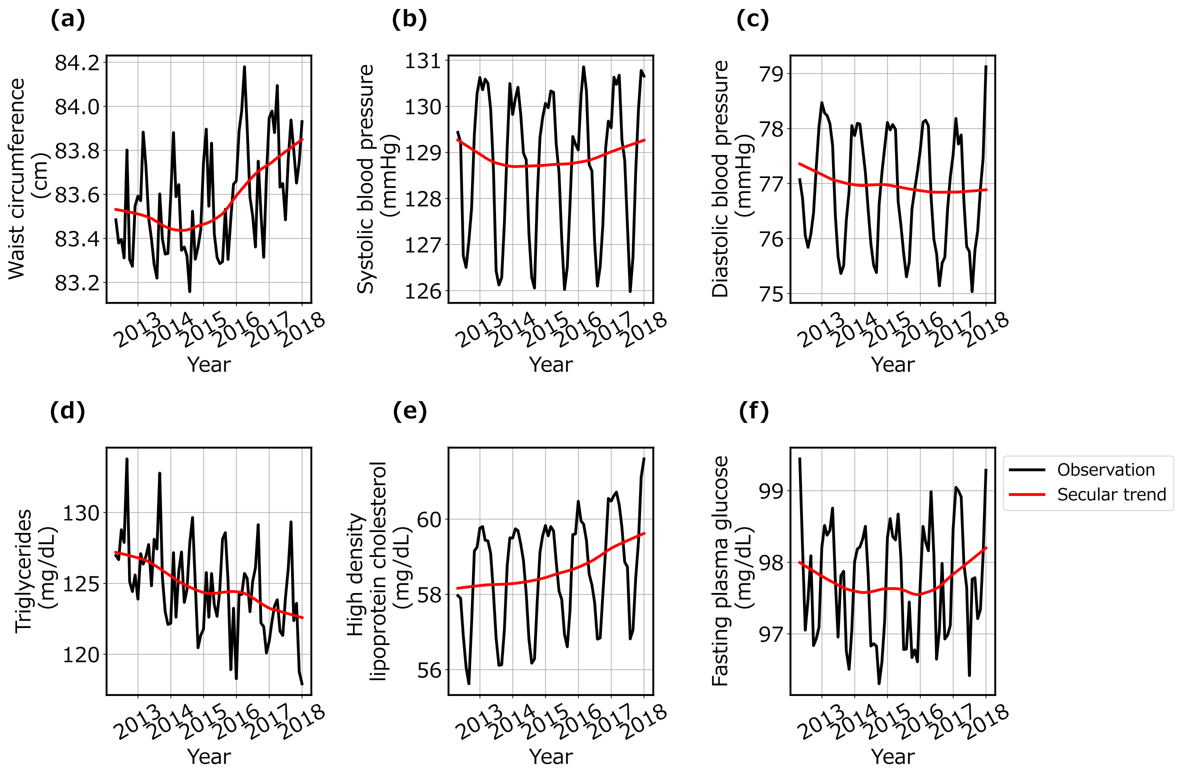
(a)
Waist circumference (cm)
84.2
84.0
83.8
83.6
83.4
83.2
(b)
Systolic blood pressure (mmHg)
131
130
129
128
127
126
(c)
Diastolic blood pressure (mmHg)
79
78
77
76
75
(d)
Triglycerides (mg/dL)
130
125
120
(e)
High density lipoprotein cholesterol (mg/dL)
60
58
56
(f)
Fasting plasma glucose (mg/dL)
99
98
97
2013
2014
2015
2016
2017
2018
Year
Observation
Secular trend

Fig. 6

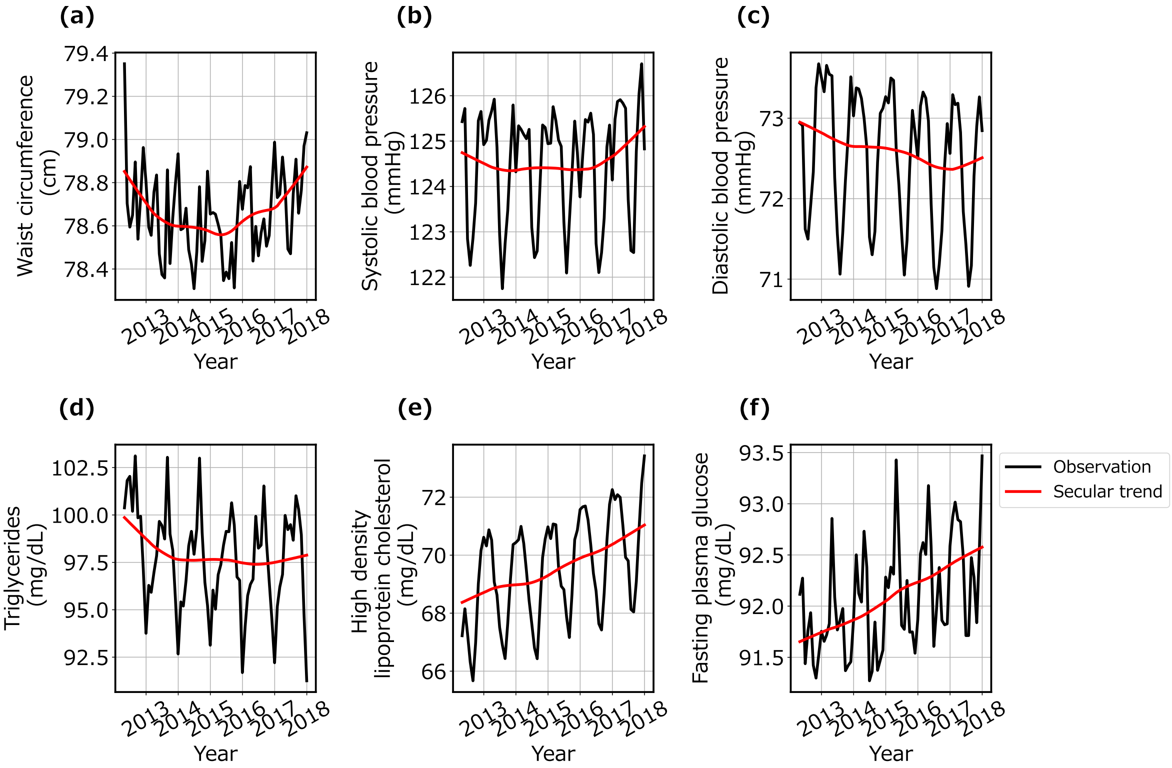

(a)
(b)
(c)
(d)
(e)
(f)
Waist circumference (cm)
Systolic blood pressure (mmHg)
Diastolic blood pressure (mmHg)
Triglycerides (mg/dL)
High density lipoprotein cholesterol (mg/dL)
Fasting plasma glucose (mg/dL)
Year
Observation
Secular trend

Fig. 7

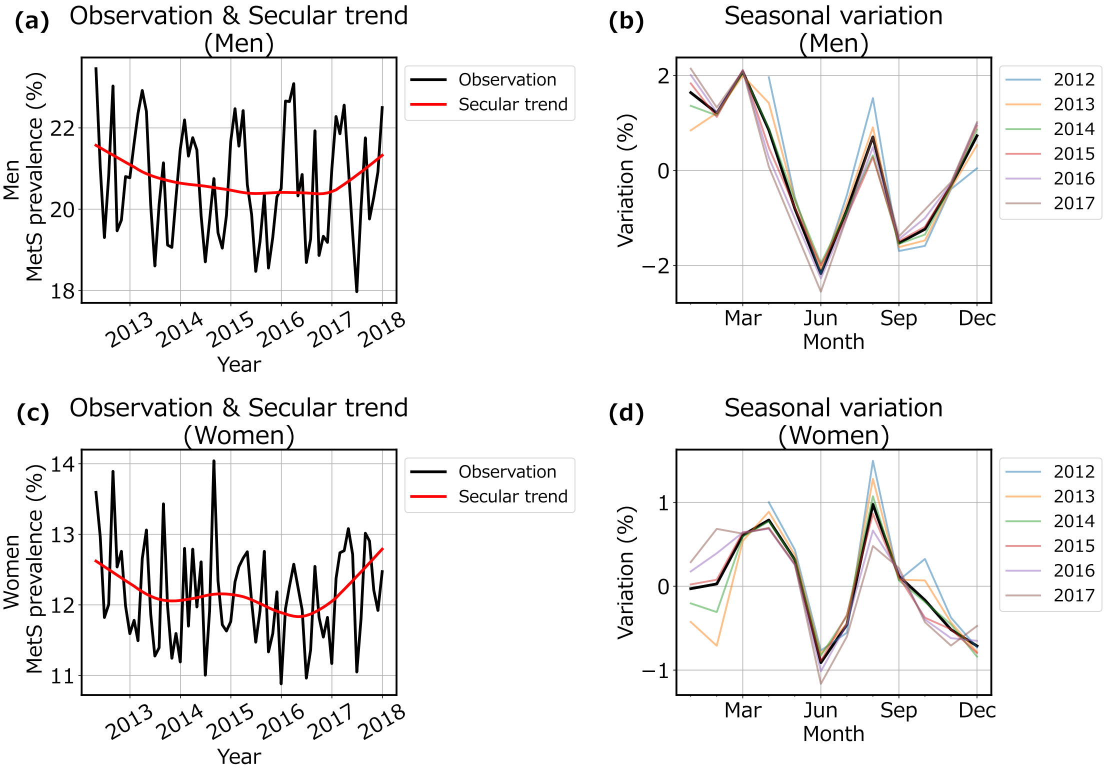
(a)
Observation & Secular trend
(Men)
Men
MetS prevalence (%)
Observation
Secular trend
Year
(b)
Seasonal variation
(Men)
Variation (%)
Month
2012
2013
2014
2015
2016
2017
(c)
Observation & Secular trend
(Women)
Women
MetS prevalence (%)
Year
(d)
Seasonal variation
(Women)
Variation (%)
Month

Fig. 8

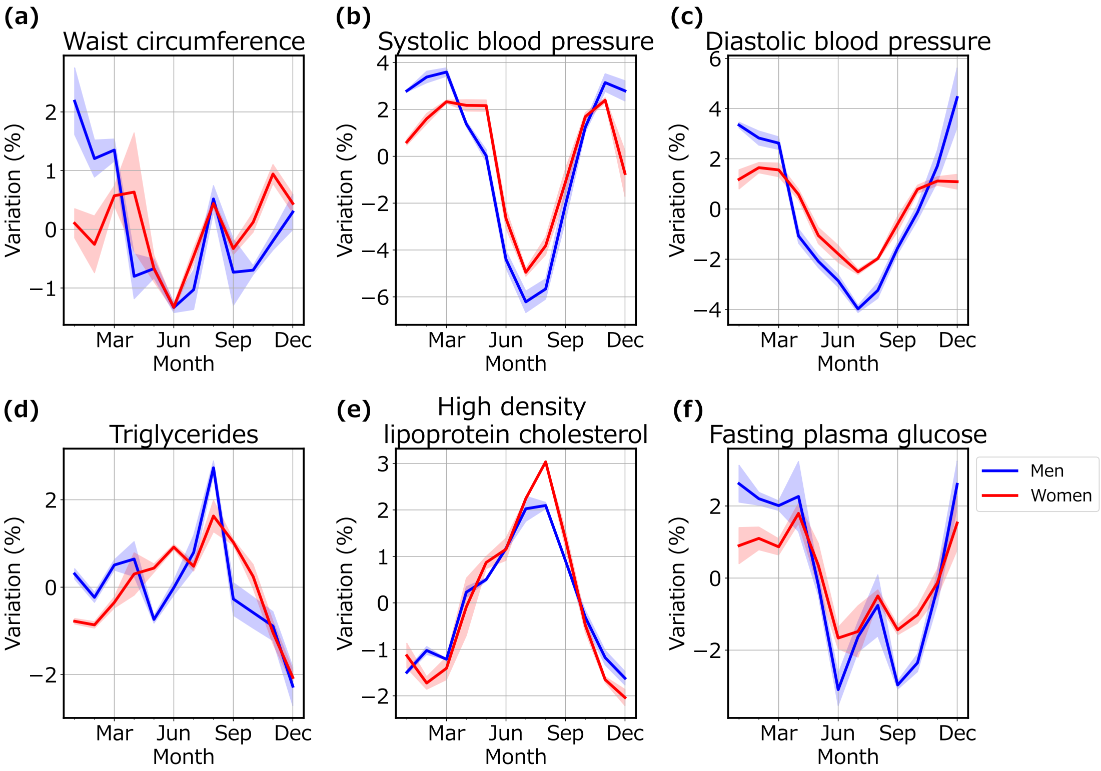

(a)
Waist circumference
Variation (%)
Mar
Jun
Sep
Dec
Month
(b)
Systolic blood pressure
(c)
Diastolic blood pressure
(d)
Triglycerides
(e)
High density
lipoprotein cholesterol
(f)
Fasting plasma glucose
Men
Women

Fig. 9
